# Protective Effects of Exercise Become Especially Important for the Aging Immune System in The Covid-19 Era

**DOI:** 10.14336/AD.2021.1219

**Published:** 2022-02-01

**Authors:** Katarzyna Domaszewska, Michał Boraczyński, Yi-Yuan Tang, Joanna Gronek, Krystian Wochna, Tomasz Boraczyński, Dariusz Wieliński, Piotr Gronek

**Affiliations:** ^1^Department of Physiology and Biochemistry, Poznan University of Physical Education, Poland.; ^2^Faculty of Health Sciences, Collegium Medicum, University of Warmia and Mazury in Olsztyn, Poland.; ^3^College of Health Solutions, Arizona State University, USA.; ^4^Laboratory of Genetics, Department of Dance and Gymnastics, Poznan University of Physical Education, Poland.; ^5^Laboratory of Swimming and Water Lifesaving, Faculty of Sport Sciences, Poznan University of Physical Education, Poland.; ^6^Faculty of Health Sciences, Olsztyn University, Poland.; ^7^Department of Anthropology and Biometry, Poznan University of Physical Education, Poland.

**Keywords:** aging process, exercise, immune system, immunosenescence, COVID-19

## Abstract

Aging is a complex, multietiological process and a major risk factor for most non-genetic, chronic diseases including geriatric syndromes that negatively affect healthspan and longevity. In the scenario of “healthy or good aging”, especially during the COVID-19 era, the proper implementation of exercise as “adjuvant” or “polypill” to improve disease-related symptoms and comorbidities in the general population is a top priority. However, there is still a gap concerning studies analyzing influence of exercise training to immune system in older people. Therefore, the aim of this review is to provide a brief summary of well-established findings in exercise immunology and immunogerontology, but with a focus on the main exercise-induced mechanisms associated with aging of the immune system (immunosenescence). The scientific data strongly supports the notion that regular exercise as a low-cost and non-pharmacological treatment approach, when adjusted on an individual basis in elderly, induce multiple rejuvenating mechanisms: (1) affects the telomere-length dynamics (a “telo-protective” effect), (2) promote short- and long-term anti-inflammatory effects (via e.g., triggering the anti-inflammatory phenotype), 3) stimulates the adaptive immune system (e.g., helps to offset diminished adaptive responses) and in parallel inhibits the accelerated immunosenescence process, (4) increases post-vaccination immune responses, and (5) possibly extends both healthspan and lifespan.

Aging is a complex, multietiological process and a major risk factor for most non-genetic, chronic and non-communicable diseases (NCDs) including geriatric syndromes that negatively affect healthspan and longevity [1, 2, 3]. Age-related disorders account for ~25% of the global burden of disease and continue to increase [4]. From a biological perspective, aging is associated with cellular senescence. This specific irreversible processwas first identified by Hayflick and Moorhead [5] who discovered that diploid cells (fibroblasts) invariably undergo a finite and predictable number of cell divisions due to telomere shortening. This process was termed as “replicative senescence”. In subsequent years, the telomere length-independent senescence was described in detail by Roy Walford [6], known as the immunologic theory of aging. According to his classical book, age-related pathological phenomenon defined as “immunosenescence” leads the immune system to a progressive reduction in the ability to trigger effective cellular and antibody responses against the virulence of infectious diseases and vaccinations. It also affects both ancestral/innate and acquired/adaptive immunity, although T lymphocytes are dramatically affected [6]. However, age-related immunosenescence is more extensively influenced by acquired immunity than innate immunity [7].

In general, human immunosenescence may be influenced by several factors inlcuding genetics, exercise, nutrition, previous exposure to microorganisms, biological sex, persistent human cytomegalovirus (HCMV) and Epstein-Barr virus (EBV) infectionsand other environmental factors such as air pollution [8-12]. Additionally, some processes seem particularly important for aging in the context of severe acute respiratory syndrome coronavirus 2 (SARS-CoV-2) pandemic. First, older adults with immunosuppressed or weak immune defense are susceptible to coronavirus disease 2019 (COVID-19), caused by SARS-CoV-2. Recently there have been more concerns directed toward this group since the latest studies confirm a dramatic decrease in their engagement in physical activity (PA) in outdoor green settings [13, 14]. It is important to note that physical inactivity is the 4th most important risk factor for global mortality [15] and sedentary time results in limited non-exercise physical activity (NEPA) of daily life [16]. Unfortunately, ~1/3 of adults worldwide are currently physically inactive [17], which got further exacerbated by COVID-19 and resulted in pandemic sedentariness/ physical inactivity. This trend, as an effect of various restrictions aimed at reducing social and physical contact, is especially dangerous for patients who had been prescribed therapeutic exercise programs. Second, the public health service has been ineffective in many countries since the surge of the COVID-19 pandemic and older adults have died due to the inability to be admitted to hospitals and many suffered from an acute or chronic condition that can normally be treated, such as strokes and heart attacks. Moreover, according to recent data, COVID-19 should be referred to as a “gerolavic” (from Greek, géros “old man” and epilavís, “harmful”) disease, because the global statistics show that substantially more severe symptoms and lethality occurs in the population aged >60 years old [18].

Additionally, the immunopathological mechanisms of COVID-19 and associated death rates are still unclear. It is predicted that by 2050 there will be 40% of people over the age of 60 in Poland [19], and globally, the coming “tsunami” of aging of societies are also expected. Thus, the percentage of older people in the structure of society turns the age pyramid.

These circumstances force health and exercise specialists in PA to realize that regular physical exercise (even in a form of home-based exercise) have not only affect positive body image, sexual desirability, well-being and “endorphins”, but it becomes a matter of survival for the older population in the COVID-19 era.

Therefore, the aim of this review is to provide a brief summary of well-established findings in exercise immunology and immunogerontology, but with a focus on the main exercise-induced mechanisms associated with aging of the immune system (immunosenescence).

## 1.Exercise and immunity

In general, exercise (a part of PA) is defined as a planned and structured behaviour, which aims to improve specific health and physical performance outcomes, such as functional ability or cardiorespiratory fitness [20]. Exercise can be classified into four subclasses: resistance, endurance, cognicise (*cogni*tive and exer*cise*) [21,22] and patterned movements [23]. Resistance, endurance and cognicise exercise are recognized as stimualtion of the body that has a significant influence on skeletal muscle tissue, nervous system and a more youthful immune phenotype [12]. In turn, patterned movement exercises concern mainly a motor program in the central nervous system and result in relatively non-signifficant changes in muscles physiology [23]. Thus, the following questions arise:

1. Can regular PA ameliorate immunosenescence and thereby reduce age-related multi-morbidity?
2. What kind of PA and which mode best fits for elderly in the context of immunosenescence?

Regular PA is recommended by World Health Organization [15] due to well-established association with increased longevity as a consequence of a reduced risk of developing cardiovascular diseases (CVDs), neuro-degenerative diseases, type 2 diabetes mellitus (T2D), metabolic syndrome, hypertension, infectious diseases and cancer [24-27]. Studies focused on analysing the effects of exercise training interventions on immunity in older people usually adopt a longitudinal randomized controlled trials (RCTs) to document immune changes in response to an intervention ora cross-sectional model to discriminate between active and inactive subjects [28]. The most frequent exercise training intervention protocols analyze aerobic or resistance exercise (or a combination of both, *i.e.*, concurrent strength and endurance training) ranging from 8-weeks to 12-months. However, there is still a gap concerning studies analyzing influence of cognicise training to immune system in older people.

The most common outcome data concerned: 1) T-cells: subset numbers, mitogen-induced T-cell proliferation, phenotype characteristics, 2) serum antibody titres following vaccination, 3) cytotoxic activity of NK-cells, and 4) neutrophil/monocyte phagocytic function [29]. For instance, the amount of PA required to maintain neutrophil/monocyte phagocytic function equates to ~10,000 steps/day [30].

However, more often than not, the comparative analysis of the results among the longitudinal studies is complicated since different exercise protocols have been applied including different modes, volume, intensity, and frequency of a single bout. Additionally, age of participants varies between experiments and in some studies, it is not clear what exclusion criteria were adopted. Although in this review we have focused literally on the relationship between exercise and the immunosenescence, we must not forget about the broader consequences well beyond the effects on the immune system of exercise in the elderly. This means that in order for the analysis not to be purely theoretical, we must not overlook the other needs of the aging organism, such as plasticity of nervous system, general cardiorespiratory fitness and other important variables during healthy aging.

## 2.Exercise and aging of the cell

### Telomeres

Telomeres are stretches of repetitive DNA (5′-TTAGGG_n_-3′) sequences that cap eukaryotic chromosomal ends guarding genomic DNA from enzymatic degradation and are shortened progressively with each cycle of mitosis. Currently, leukocyte telomere length (LTL) is one of the most accepted markers of biological aging since Njajou et al. [31] observed strong possitive association between LTLwith the number of years of healthy living. Thus, a shorter LTL is recognized to be older than longer ones [32]. It has been proven that average telomere length diminishes from 11 kilobases after birth [33] to less than 4 kilobases at an older age [34]. Senescence is triggered when the telomeric terminal restriction fragment (TRF) reaches length of 4-7 kilobaseson average [35].

Moreover, LTL is influenced not only by numbers of succesive mitosis but also by other factors such as gender, BMI, smoking history, leisure time and PA, even after adjusting for age [36]. The study on 2401 white twins (2152 women and 249 men) also reported that LTL was even 200 nucleotides longer in physically active individuals compared to age-matched sedentary subjects [36]. This value corresponds to ~10 years of estimated biological aging. Several subsequent studies confirmed such relationships [37, 38]. More detailed information concerning PA and telomeres was shown by Ludlow et al. [39], who reported that individuals expending less than 990 kcal per week had shorter leukocyte telomeres than subjects expending between 991 and 2340 kcal per week, however no differences were observed for telomerase activity, which synthesizes telomeric DNA repeats. Recently, Simões et al. [40] presented data suggesting that longer LTL and higher physical fitness protect master athletes from severe consequences of SARS-CoV-2 infection.

While numerous studies indicate association between LTL, aging and PA, such positive associations have been refuted by several observational and interventional studies [see review, 41]. Nevertheless, the proposed associations of LTL with aging and exercise are biologically plausible. However, the mechanism explaining how training and PA affect LTL has still not yet been discovered [42, 43]. Overall, the presented data indicate a potential “telo-protective” effect of exercise on leukocytes, although it also applies to other cells (e.g., cardiac and liver cells) [44].

### Oxidative stress

Reactive oxygen species (ROS), including superoxide anion (O2-), hydrogen peroxide (H2O2), and hydroxyl radical (HO•), consist of radical and non-radical oxygen species formed by the partial reduction of oxygen. The generation of cellular ROS are produced by immune cells - neutrophils and macrophages - as a result of increased aerobic metabolism during vigorous immune responses (the process of respiratory burst) and are considered as an important defense mechanism against pathogens [45]. Free radicals are unstable atoms that are considered as the “prime suspect” of aging processes and an actual source that contribute to cellular senescence, at least partially [46]. This approach was first outlined by Denham Harman in 1956 [47].

Exercise leads to increased oxidative stress (Ox stress). However, exercise-related oxidative damagealso seems necessary to trigger the adaptive up-regulation of molecular and cellular pathways (including endogenous antioxidant defense mechanisms) according to the hormesis theory [48, 49].

The hormesis concept, proposed for the first time by Southam and Ehrlich [50], assumes that in contrast to the obvious toxic effect of high doses of certain harmfull environmental factors (materials, irradiation, Ox stress, etc.), low doses of them tend to be beneficial and, moreover, help to eliminate (or prevent) the deleterious effect of high doses exposed after it [51]. Some authors emphasise that hormesis is an important factor of (human) evolution protecting human from harmful impacts, similar to the role of immunity [52, 53]. Specifically, hormesis hypothesis suggests that the organism’s response to amplified increases in ROS production via exercise bouts triggers adaptive mechanisms [54].

Adaptive mechanism enhances an antioxidant upregulation including catalase (CAT), glutathione peroxidase (GPx), superoxide dismutase (SOD), glutathione reductase, glutathione-S-transferase, as well as nonenzymatic antioxidants (vitamins A, E, C) that protects the body against excessive oxidative stress related to aging. A number of studies proved the hypothesis that ROS are required for the health-promoting effects of exercise, causing an increase in endogenous antioxidant defense mechanisms (via up-regulated expression of antioxidant genes) and consequently, prolong healthspan and mean lifespan [55, 56]. However, as the relationship between oxidative stress and PA is still poorly explained, particularly in advanced age, at least two questions arise here concerning two main exogenous adaptive factors: antioxidants and exercise. First, is it really a common supplementation of antioxidants a better approach than well-balanced diet rich in fruits, vegetables and fiber, which may act in synergy to optimize the antioxidant effect [54]? Second, how can we detect or estimate which volume, intensity, frequency, and exercise loads are beneficial in the release of antioxidant mechanisms, and determine which PA load has a negative and devastating effect on the body?

## 3.The effects of exercise on the adaptive immune system

Acquired immunity of the organism is related to the response of the T and B lymphocytes to an antigen and it generally lasts for 2 up to 5 days, while repeated exposure of the organism to a pathogen produces immune memory [57]. According to Littman [58], the T lymphocytes may be further divided into subclasses depending on the expression of the CD4 and CD8 molecules on the cell surface. The T CD4+ lymphocytes recognise antigens as presented by major histocompatibility complexes (MHC) class II; these are typically the T helper (Th) lymphocytes. The other subclass comprises the T CD8+ lymphocytes. They recognise the MHC class I and are mainly the Tc lymphocytes, which are responsible jointly with cytotoxic cells for the destruction of cancer cells as well as cells infected by microorganisms [58]. They coordinate acquired immune response through the PAMP complex and dendritic cells secreting cytokines (CCL3, CXCL9, CXCL10) e.g., in the respiratory tract [59]. The molecular response pattern to pathogen infection, described by Müller et al., are involved in the inhibition of virion replication or destruction of the infected structure. Thus, the joint response of T CD4+ and CD8+ lymphocytes protects against respiratory diseases caused e.g. by influenza A or parainfluenza viruses. In healthy individuals during resting state, the CD4:CD8 ratio does not drop below 1.0 [60]. Physical activity of excessive intensity in relation to the organism’s capacity, regardless of endurance or resistance exercise, leads to a reduction of the CD4:CD8 lymphocyte subpopulation to below 1 [61]. In older people a decreased blood level of T lymphocytes including its subpopulations has been noticed [62]. Linton and Thoman and Phillips et al. explained this phenomenon among other things by the age-related thymus atrophy, as well as an increased concentration of antigen-committed memory T cells and a decrease in the level of naïve T cells capable of responding to new antigens [62,63]. Compared to younger individuals, the frequency of T lymphocytes type KLRG1+, CD57+ and CD28- is also greater in older people [64-66]. Moreover, a reduced proliferation, secretion and signalling capacity, as well as an increased apoptotic lymphocyte capacity were shown in older people [67, 68, 69]. For this reason, the overall immune performance of the organism is decreased with age. High mortality caused by infectious diseases, influenza or pneumonia has been observed in the population of older people. A study in 2020 concerning immune changes in the acute phase of the SARS-CoV-2 infection showed that in 80% of all cases, a decrease of CD4+ and CD8+ T lymphocyte subpopulation is observed along with an increase in blood proinflammatory cytokines [70]. Thus, methods that reduce inflammatory response, modulate oxidative stress, and increase the synthesis of nitric oxide (NO), can all enhance the immune system and may be helpful in the control of many diseases, including the SARS-CoV-2 infection [71-72].

Numerous factors affect the performance of the immune system. To date, research has shown the effect of physiological factors, nutrition, psychological factors and environmental conditions on the rate of which immune performance deteriorates with age. Regular, moderate exercise will help boost the immune system at any age, thus reducing the risk of incidence or severe course of many diseases [73]. Just 10 consecutive days of endurance exercise contributes to significant protection against respiratory dysfunctions [74]. Exercise promotes apoptosis of aging T lymphocytes and stimulates synthesis of new, fully functional immune cells. Although the total number of lymphocytes and the subpopulation of the T lymphocytes released to blood during exercise probably is not age-dependent, the phenotypes of mobilised cells vary among individuals in different age groups [75]. Some of the positive effects of regular exercise are related to an increased proliferation capacity of the T lymphocytes, the activity of neutrophils and cytotoxic activity of the NK cells. This change is dependent on the level of physical fitness, duration of exercise, its intensity and particularly acidification of the organism. The leukocyte count increases with an increase in the intensity of exercise and blood lactate levels. In accordance with the generally known phenomenon of hormesis, which as an adaptive response explains both the advantageous and disadvantageous effects of exercise. In particular the lymphocyte subtypes of high cytotoxic potential (i.e., the NK cells, the T CD8+ cells) are more sensitive to stress induced by exercise compared to the T CD4+ lymphocytes or the B lymphocytes, which exhibit limited cytotoxic capacity and are mobilised in a relatively lower number [76]. In a situation when effort is made, considerably exceeding the physical fitness capacity of the organism, it leads to leukopenia, deregulation of inflammatory processes and reduced capacity to maintain homeostasis and particularly the reduction of systemic immunity. Also, in the period of post-exercise regeneration of the organism, a transitional lymphocyteopenia lasting for 6-24 h is observed [77]. This is probably caused by increased apoptosis or migration of cells outside the vascular area. Both these mechanisms may overlap. Thus, this condition is called by many authors “the open window” [78]. The mechanisms underlying exercise-induced changes in the immune system include among other things changes in the concentration of neurohormonal factors depending on the intensity of exercise (e.g., cortisol, catecholamines, the growth hormone, endorphins, sex steroids), specific immune parameters (e.g., mucosal lactoferrin, immunoglobulin A) and cytokines (e.g., TNF, IL-1, IL-6) [79]. Additionally, changes in blood concentrations of glutamine, glucose, lipids or thermal shock proteins contribute to the incidence and development of leukocytosis. In the initial phase of exercise, the increase in the number of leukocytes is caused by the action of catecholamines, whereas a successive, already slower increase results from the effect of cortisol on the bone marrow. It is known that cortisol has an immunosuppressive effect. It may thus inhibit the function of cytokines and the NK cells, as well as reduce the production of the T lymphocytes during high intensity exercise.

## 4.Exercise and viral infections

The intensity of exercise plays an important role in the clinical resistance to infections. Short-term exercise of low intensity, as recommended by American College of Sports Medicine [80] for individuals of a low physical fitness level, has a slight effect on changes in blood antibody concentrations. Martin *et al*. [81] put forward a hypothesis that it is regular exercise in moderate intensity and duration that leads to an increase in stress hormone concentrations at any age, at the same time reducing inflammation in the airways, thus promoting activation of innate antiviral resistance. From this perspective, such exercise is associated with a reduced incidence, duration and severity of upper respiratory tract infections (URTIs), especially respiratory viruses as influenza and rhinovirus [82]. The cellular and molecular mechanisms underlying this phenomenon are related with changes in the intensity of the immune response reflected in the T helper 1/T helper 2 (Th1/Th2) ratio. The primary role of the Th1 lymphocytes includes their participation in the cellular response reactions, as they exhibit a proinflammatory action in viral and protozoan infections as well as cancer. Active substances released by this type of lymphocytes include interleukin 2 (IL-2), interferon gamma (IFN-γ) and tumour necrosis factor α (TNF-alfa). The Th2 lymphocytes play a key role in humoral immune responses. Characteristic compounds produced by the Th2 lymphocytes include anti-inflammatory interleukins 4 (IL-4), IL-5, IL-9, or interleukin 13 (IL-13). The interdependence between these types of lymphocytes have been described. Cytokines produced by Th1 have an adverse effect on the development of the Th2 cells and vice versa. The described phenomena underlie the immune polarization, and they serve a crucial role in the regulation of immune response. Physical activity of moderate-intensity causes a slight increase in blood concentrations of glucocorticosteroids, catecholamines or IL-6 leading to a slight deviation in the Th1/Th2 ratio towards Th2. Thus, they determine an appropriate development of adaptive immune response. Very high-intensity exercise results in a considerable increase in blood concentrations of glucocorticosteroids and catecholamines, causing a marked shift in the Th1/Th2 ratio towards Th2. This leads to a reduction of proinflammatory response by a decrease in the function of effector cells (Mϕ, NK) and an inappropriate development of adaptive immune response [81, 83]. Existing studies mostly concern the efficacy of regular PA in controlling the course of respiratory viral diseases in an animal model and in humans [84, 85]. At the same time, experimental studies on animals have shown the efficacy of PA of varying intensity as a protective mechanism against infection or infection symptoms, as well as mortality due to respiratory system viruses [86, 87]. Unfortunately, application of excessively intensive exercise resulted in increased morbidity and mortality rates caused by viral respiratory infections [88]. A similar mechanism is likely found in humans. In view of the high annual incidence of influenza given the current healthcare system overload, it is highly advisable to maintain health-promoting PA in combination with vaccinations provided by the public healthcare system. Chubak et al. [89] in their study assessed the effect of moderate-intensity exercise on the development of common cold symptoms in obese, physically inactive postmenopausal women. Based on 12-month observations those researchers stated that exercising individuals reported a much lower number of cold symptoms compared to the physically inactive controls [89]. In contrast to numerous studies showing efficacy of exercise in the prevention of viral infections, a study by Weidner et al. [90] indicated a lack of efficacy of moderate exercise in reducing the incidence of rhinovirus-caused upper respiratory illness (URI) among vaccinated and non-vaccinated individuals. A possible limitation in this case may be related to the relatively high baseline level of physical fitness in the participants prior to the experiment. For ethical reasons, no data are available in literature on the efficacy of PA in a similar experimental model, where participants have more serious viral infections caused by the influenza virus, adenoviruses or enteroviruses.

Thus, according to Momesso dos Santos et al. [91], it is moderate-intensity exercise that may have an anti-inflammatory and immunomodulatory effect. In view of the above-mentioned phenomena, exercise may be the safest form of immunotherapy both in children and adults [91]. Moreover, it was also shown that extended exercise enhances the immune response against bacteria and viruses. Considering the currently high morbidity and mortality, particularly among older people due to SARS-CoV-2, a regular individualised training programme in the period before the development of pathological changes may protect against serious consequences of the infection. To date, there has not been studies on the effect of PA on immune performance of the organism infected by SARS-CoV-2.

It may be assumed that inactivity influences not only the course, but also the severity of many comorbidities, which significantly increases mortality rates caused by infection [92]. Physically active muscles release cytokines, which are capable of counter the proinflammatory mediators, *i.e.*, IL-1β and IL-18, at the same time increasing the concentration of interleukins, TGFβ, IL-1rα and IL-10, which enhance the anti-inflammatory effect of the immune response [93, 94]. During PA the secretion of hormonal inhibitors of cytokines (cortisol, prostaglandins, soluble receptors against TNF and IL-2) is also modified or the expression of TLR4 is inhibited, thus facilitating control of inflammatory conditions in patients suffering from chronic diseases [95]. In the course of a severe form of the COVID-19 disease, an overproduction of cytokines and immune cells takes place (“cytokine storm”), causing dangerous arterial hypertension, damage to the lungs or other internal organs. For this reason, the role of PA to reduce the inflammation and increase synthesis of anti-inflammatory cytokines, postulated in many publications, may play an important role in the course and prognosis in SARS-CoV-2 infections.

## 5.Exercise and vaccine efficacy

Upon neutralisation of the antigen by the immune system, most cells undergo apoptosis. The other cells remain alive and become the so-called antigen-specific memory cells. As a result, in the future when exposed to the same antigen they are capable of a prompt response. The described mechanism of generating immune memory cells constitutes the basis for vaccine efficacy. Multicentre randomised studies within the last 15 years have shown that exercise taken both before and after vaccination induce a higher antibody titre to the administered antigen. Those studies concerned the efficacy of vaccinations against influenza, tetanus, meningitis, diphtheria, meningococci or pneumococci [96-98]. Most presented studies were conducted on young individuals with a relatively strong antibody response to vaccination. In this group an additional intervention provided by PA did not contribute to an increased antibody titre, while efficacy of vaccination in prevention of hospitalisation amounted to approx. 75%; unfortunately, it dropped to 45% and 30% in individuals aged 65 and 75 years, respectively. This suggests that the older population exhibiting a decreased antibody response to vacciations may experience greater benefits from the exercise intervention than populations with greater immunity [99]. In another study the immunoglobulin M (IgM) and immunoglobulin G (IgG) antibody titres and immune response to a flu vaccine were assessed in the period of 2 weeks after immunisation. It was shown that a higher titre of antibodies was recorded in physically active older adults [100]. In line with this finding, it was showed that a 3-month cardiovascular exercise training extends influenza (H3N2 variants) vaccine seroprotection in sedentary older adults [101]. Unfortunately, prophylactic vaccination is less effective in older population, especially in those who are frail [102]. Therefore, it seems highly promising to apply regular PA to older people, who will be vaccinated against SARS-CoV-2 in the nearest future.

## 6.Exercise and age-associated inflammation

Inflammaging, influenced by immunosenescence, is a term proposed by Franceschi et al. [103] and is the combination of “inflammation + aging” to indicate the chronic, low-grade inflammation status is interconnected with the aging process. Inflammaging is believed to be a consequence of a cumulative lifetime exposure to antigenic load and stress, associated with continous inflammatory stimuli representing a biological background which leads to age-related diseases [104, 105].

However, do we really have a problem with inflammaging per se or maybe this pathological aging mechanism should be rather explained by “inflamm-inactivity”? The latter term constitutes a new paradigm introduced by Flynn et al. [106] to highlight exercise as a major component in the inflammaging model. In this context, can we assume that inflammaging is escapable? If the answer is positive, it would mean that the key solution is in our hands. In other words, to control and moderate the effects of immunosenescence and inflammaging, we need to focus on the manipulation of certain lifestyle factors, like increasing PA levels [107]. It remains unclear whether exercise can prevent or transiently reverse these hallmarks of aging process, but in this area, we have a large evidence-based data of positive impact of vary and chronic exercise training interventions [12, 108, 109, 110]. Indeed, one of the highly effective non-pharmacological strategies that reduce age-related inflammation and the associated diseases within the “diseasome of physical inactivity” [111], and at the same time improve the quality of life (QoL) in older adults is regular, planned exercise [112]. In the scenario of “healthy or good aging”, especially during the COVID-19 era, the proper implementation of exercise as “adjuvant” or “polypill” to improve disease-related symptoms and comorbidities in the general population is a top priority [17].

Regular, long-term exercise training is a major geroprotective intervention as it ameliorates immune function, whereas a prolonged period of sedentary lifestyle accumulated over weeks, months or years (activities that require low energy expenditure, e.g., sitting: <1.5 METs - metabolic equivalents; 1 MET = 3.5 mL O2·kg^-1^·BM·min^-1^) leads to immune supression [113] and hyper-inflammation state [106, 114]. The study on exceptional longevity in men provided information that vigorous exercise was associated with approx. 20-30% decreased mortality risk before age 90 years in a cohort of 2357 men (mean age was 72 years, range 66-84 years) [115]. A number of previous scientific reports have demonstrated an inverse dose-response relationship between systemic inflammation (e.g., circulating C-reactive protein, CRP) and exercise training and fitness status [116, 94, 117]. This dose-dependent effect is a multi-factor product of underlying mediators: the mode, duration, intensity, and frequency of exercise, that accumulate in a lifetime exposure [12]. This is due to different types of exercise evoke significant short-term (the minutes and hours after a single bout of exercise), medium-term (e.g., 1-3 weeks) and long-term (e.g., months or years of regular structured exercise) anti-inflammatory effects both in women and men, irrespective of age [93, 118, 111]. For example, among people 70-79 years old enrolled in the Health, Aging and Body Composition (ABC) study, trends for decreased cytokine concentrations (IL-6, TNF-α and CRP) were linear with increasing amounts of reported exercise [94]. The exercise-induced anti-inflammatory mechanisms control and resolve the inflammatory processess and inhibit age-related pathologies in many ways [119]. In healthy aging, the specific variants of regular exercise (particularly aerobic exercise training, AET, or cardiovascular exercise, e.g., walking, running, cycling): acute bouts of moderate-intensity (3-6 METs or 50-69% of maximal oxygen consumption; V?O_2max_) and vigorous-intensity exercise (>6 METs or ≥70 % V?O_2max_) when achieving moderate-to-high peak cardiorespiratory fitness, reduce the risk of CVD and all-cause mortality [27]. These types of exercise also appear to positively affect the immune system, enhancing adaptive responses and reducing chronic inflammation [29, 118]. Practically, the recent recommendations for immunoprotective exercise are as follows: intensity of exercise should be 60-75% of HR_max_ or 50-60% of V?O_2max_ with rating of perceived exertion (RPE) of 10-14/20, frequency of exercise being 3-5 days/week and sessions ranging from 20 to 60 min [108]. Moreover, 75 minutes per week of specific high-intensity interval training (HIIT, with single session up to 10 min) is considered to be an effective strategy to achieve optimal functionality of the immune system in the elderly [120]. In the framework of HIIT also resistance training (e.g., bodyweight exercises) with sets of short exercise (< 1 min) or low number of repetitions (< 12 reps per set) and higher speeds of movement would be beneficial in older individuals [121]. However, a relatively high individual variability of the immune response to exercise-induced stress was observed [122]. The recommended strategy to reduce exercise-induced physiological stress in the elderly is to use longer recovery intervals in relation to the stimuli (work-to-rest ratios e.g., 1:2; 1:3; 1:4) [123]. Given the subjective RPE, exercising at an RPE of 11-13 is suggested for sedentary individuals, whereas an RPE of 13-15 may be recommended for those who have been previously exercising [124]. Aerobic exercise training is considered a single, hormetic stress stimulus that initiate mitochondrial biogenesis, antioxidant defense, cellular repair and recycling, and immunity [125, 49]. Recently, running was even termed the “key lifestyle medicine for longevity” [126]. Hence, according to a few large population-based cohort studies and meta-analyses, it is not surprising that the risk of CVD-related and cancer-related mortality in runners compared with non-runners (after adjusting for potential confounders) is reduced by 45-70% and 30-50%, respectively [127-130]. Additionally, running exerts protective effects on neurological health, reducing the systemic inflammation and consequently the risk of e.g., Alzheimer’s and Parkinson’s disease [127]. The problematic issue concerns running participation as it continues to decline 5-10% per decade, and less than 2% of people continue to run over 65 years of age [131].

Nowadays, the paradox of both inflammaging and immunosuppression is observed with increasing age worldwide [132]. However, the “anti-inflammaging” effects may also have some negative implications. For instance, there exists the theory that heavy exercise, termed as “dangerous exercise”, elicits an immunological “danger” type of stress which, at some timepoints, becomes dysregulated and detrimental to health status, e.g., anaphylaxis, exercise-induced asthma, overuse syndromes, and exacerbation of intercurrent illnesses [133]. This refers to the long-duration one-off bouts of exercise (e.g., marathon running or cycling), extreme forms of exercise such as ultra-endurance races (e.g., Ironman triathlon), and long-duration (i.e., weeks or months) of very high-intensity athletic training [134]. It was recognized that acute, particularly high-volume intensive exercise sessions can cause transient immunosuppression (i.e., 1-3 h post-exercise) [135]. On this theoretical background and from the available cross-sectional data, it appears that moderate intensity exercise provides anti-immunosenescence effects (expressed by i.a., a less differentiated T-cell profile), whereas high-volume intensive exercise (typical in highly competitive athletes) drives mostly pro-immunosenescence effects (expressed by i.a., a more differentiated T-cell profile, increased expression of glycoprotein and myosin levels, and reduced thymic output) [107].

**Figure 1.**
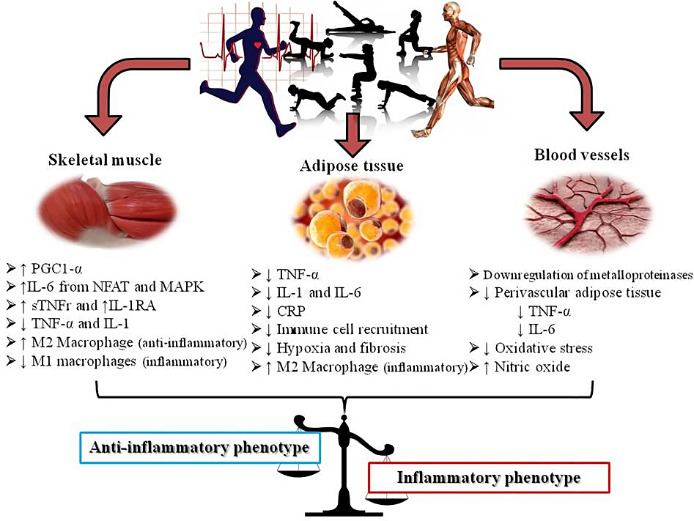
Mechanism of exercise in activation of anti-inflammatory phenotype in different tissues. PGC1α: peroxisome proliferator-activated receptor γ co-activator 1α, IL-6: interleukin 6, NFAT: nuclear factor of activated T-cells, MAPK: mitogen-activated protein kinase, sTNFr: soluble tumour necrosis factor receptors, IL-1RA: interleukin 1 receptor antagonist, TNF-α: tumour necrosis factor alpha, IL-1: interleukin 1, CRP: C-reactive protein. Figure adapted from Metsios et al. [20]

It was observed that sentinel and effector cells known as monocytes/macrophages (an important mediator of disease progression) can alter their phenotype in response to changes in the local cytokine environment [136]. In general, macrophages can be distinctly activated to either pro-inflammatory phenotype (also referred to as “M1”-like) or to anti-inflammatory phenotype (also referred to as “M2”-like) [137, 138]. Specifically, subtype 1 (M1) macrophage polarization is associated with inflammation and tissue destruction (induced by interferon-γ and/or lipopolysaccharide, LPS), whereas the subtype 2 (M2) macrophage is associated with wound repair and angiogenesis (induced by IL-4 and IL-13) [139]. An increasing body of evidence suggests that engagement in exercise induces an anti-inflammatory phenotype in the general population, both acutely as well as in the long term (Figure 1). This is true at local and systemic level, because the short-term effects of exercise on inflammation have been mainly investigated in the skeletal muscle tissue by assessing muscle fiber-derived cytokines and peptides termed “myokines”, such as IL-15, LIF, brain-derived neurotrophic factor (BDNF), irisin and fibroblast growth factor 21 (FGF-21), which increase and remain elevated during and post-exercise [111, 140, 141]. Of note, the most studied myokine IL-6 increases up to 100-fold in the circulation during physical exercise [111] or even up to 128-fold immediately after exercise compared to the pre-race value [142]. The substantial increase of IL-6 also stimulatesthe production of the classical anti-inflammatory cytokines like IL-1RA and IL-10 [143]. Importantly, older people with high levels of anti-inflammatory cytokines (especially IL-10) seem to have a healthy aging status compared to others [144]. On the other hand, the long-term effects of exercise on inflammation are mainly observed in adipose tissue with adipose tissue-derived cytokines collectively termed “adipokines” [20] or „adipocytokines” [145] (Fig. 1).

Exercise training leads to decreased white adipose tissue (WAT) inflammation, thereby ameliorating metabolic dysfunctions (*e.g.*, insulin resistance and hepatic steatosis), even in the absence of fat loss [146]. Hence, exercise-mediated reductions in WAT inflammation in humans are often weight loss-independent [147]. Nevertheless, weight loss is crucial to obtain optimal metabolic status, because it results in a substantial reduction in systemic levels of both circulating markers of inflammation (including CRP, IL-1, IL-6, IL-18, TNF-α and TNF-α receptors) and adipose-tissue cytokine production in individuals [148]. Obesity and other inflammatory degenerative diseases cause chronic inflammation silent that is without symptoms; wheras the increase in the expression of three pro-inflammatory cytokines: IL-1, IL-6 and TNF-α constitute an “inflammatory triad” - a specific hallmark of inflammaging [149]. For example, Dorneles et al. [150] found that changes in inflammatory cytokines after high-intensity interval exercise depends on a person’s body mass; the increase in cytokines (IL-6 and IL-10) is higher in obese compared to lean people. Kern et al. [151] reported that pro-inflammatory cytokine TNF-α release from abdominal subcutaneous adipose tissue was 7.5-fold higher in tissue from obese (BMI 30-40 kg/m^2^) compared to lean (BMI > 25 kg/m^2^) individuals. The authors presented also a significant relationship between TNF-α and total body fat in kilograms (*r* = 0.41, *p* < 0.05). Conversely, it is well-established that acutely promoted anti-inflammatory effects of exercise include the significant increase of soluble TNF receptors which are naturally produced inhibitors of TNF-α [150]. In this context, the results of the Sellami et al. [144] study suggest that TNF-α could be used as an early biomarker of pro-inflammatory aging in elite athletes. Interestingly, while different classes of biomolecules are responsible for the cross-talk between distant organs and cells [152], for IL-15, it is supposed to have a role in the muscle-fat cross-talk via modulation of the visceral fat mass [153]. When combining all the information provided inflammation represents a pro-longevity factor at old age and exercise is a powerful agent that keeps inflammation in check.

In conclusion, there is growing evidence on the importance of certain forms of exercise as effective geroprotective interventions. Single exercise session and longitudinal exercise promote short- and long-term anti-inflammatory effects (via e.g., triggering the anti-inflammatory phenotype), respectively, and therefore suggesting a promising neurodegenerative and infectious diseases interventional target (including SARS-CoV-2 infection and COVID-19). This low-cost and non-pharmacological treatment approach, when adjusted on an individual basis in elderly, induce multiple rejuvenating mechanisms: (1) affects the telomere-length dynamics (a “telo-protective” effect), (2) stimulates the adaptive immune system (e.g., helps to offset diminished adaptive responses) and in parallel inhibits the accelerated immunosenescence process, (3) increases post-vaccination immune responses, and (4) possibly extends both healthspan and lifespan. From the current perspective of geroscience, the healthy-functional immunity and aging is strongly influenced by lifelong training routines and should be promoted broadly.

## References

[1] Booth LN, Brunet A (2016). The aging epigenome. Mol Cell, 62(5): 728-744. 27259204 10.1016/j.molcel.2016.05.013 PMC4917370

[2] Franceschi C, Garagnani P, Vitale G, Capri M, Salvioli S (2017). Inflammaging and ‘Garb-aging’. Trends Endocrinol Metab, 28(3): 199-212. 27789101 10.1016/j.tem.2016.09.005

[3] López-Otín C, Blasco MA, Partridge L, Serrano M, Kroemer G (2013). The hallmarks of aging. Cell, 153(6): 1194-1217. 23746838 10.1016/j.cell.2013.05.039 PMC3836174

[4] Prince SA, Saunders TJ, Gresty K, Reid RD (2014). A comparison of the effectiveness of physical activity and sedentary behaviour interventions in reducing sedentary time in adults: a systematic review and meta-analysis of controlled trials. Obes Rev, 15(11): 905-919. 25112481 10.1111/obr.12215 PMC4233995

[5] Hayflick L, Moorhead PS (1961). The serial cultivation of human diploid cell strains. Exp Cell Res, 25: 585-621. 13905658 10.1016/0014-4827(61)90192-6

[6] Walford RL (1969). The Immunologic Theory of Aging. Copenhagen: Munksgaard (1969).

[7] Nikolich-Žugich J (2018). The twilight of immunity: emerging concepts in aging of the immune system. Nat Immunol, 19: 10-9. 29242543 10.1038/s41590-017-0006-x

[8] Aiello A, Accardi G, Candore G, Caruso C, Colomba C, Di Bona D, et al (2019). Role of immunogenetics in the outcome of HCMV infection: implications for ageing. Int J Mol Sci, 20:E685. 30764515 10.3390/ijms20030685 PMC6386818

[9] Caruso C, Accardi G, Virruso C, Candore G (2013). Sex, gender and immunosenescence: a key to understand the different lifespan between men and women? Immun Ageing, 10:20. 23680476 10.1186/1742-4933-10-20 PMC3737094

[10] Maijó M, Clements SJ, Ivory K, Nicoletti C, Carding SR (2014). Nutrition, diet and immunosenescence. Mech Ageing Dev, 136: 116-128. 24373813 10.1016/j.mad.2013.12.003

[11] Pawelec G (2012). Hallmarks of human “immunosenescence”: adaptation or dysregulation? Immun Ageing, 9:15. 22830639 10.1186/1742-4933-9-15 PMC3416738

[12] Turner JE (2016). Is immunosenescence influenced by our lifetime “dose” of exercise? Biogerontology, 17:581-602. 27023222 10.1007/s10522-016-9642-z PMC4889625

[13] Hasson R, Sallis JF, Coleman N, Kaushal N, Nocera G, Keith N (2021). COVID-19: Implications for Physical Activity, Health Disparities, and Health Equity. Am J Lifestyle Med, 15598276211029222. 10.1177/15598276211029222 PMC9283961 35855783

[14] Lim S, Kong APS, Tuomilehto J (2021). Influence of COVID-19 pandemic and related quarantine procedures on metabolic risk. Prim Care Diabetes, 15(5):745-750. 34312109 10.1016/j.pcd.2021.07.008 PMC8286866

[15] World Health Organization. Global health risks: Mortality and burden of disease attributable to selected major risks. Geneva: World Health Organization (2009)., p. 62.

[16] Ekblom-Bak E, Ekblom B, Vikström M, de Faire U, Hellénius ML (2014). The importance of non-exercise physical activity for cardiovascular health and longevity. Br J Sports Med, 48(3): 233-238. 24167194 10.1136/bjsports-2012-092038

[17] Fiuza-Luces C, Garatachea N, Berger NA, Lucia A (2013). Exercise is the real polypill. Physiology, 28(5): 330-358. 23997192 10.1152/physiol.00019.2013

[18] Zhavoronkov A (2020). Geroprotective and senoremediative strategies to reduce the comorbidity, infection rates, severity, and lethality in gerophilic and gerolavic infections. Aging (Albany NY), 12(8): 6492. 32229705 10.18632/aging.102988 PMC7202545

[19] Statystyczny R (2016). Statistical yearbook of The Republic of Poland. Statistical Publishing Establishment, 2016.

[20] Metsios GS, Moe RH, Kitas GD (2020). Exercise and inflammation. Best Pract Res Clin Rheumatol, 101504. 10.1016/j.berh.2020.101504 32249021

[21] Gronek P, Adamczyk J, Celka R, Gronek J (2021). Cognicise - a new model of exercise. Trends in Sport Sci, 28(1): 5-10.

[22] Suzuki T, Makizako H, Doi T, Park H, Lee S, Tsutsumimoto K, et al (2015). Community-Based Intervention for Prevention of Dementia in Japan. J Prev of Alzheimers Dis, 2(1): 71-76. 29234778 10.14283/jpad.2015.42

[23] Baar K (2006). Training for endurance and strength: lessons from cell signaling. Med Sci Sports Exerc, 38(11):1939-44. 17095927 10.1249/01.mss.0000233799.62153.19

[24] Barlow CE, LaMonte MJ, Fitzgerald SJ, Kampert JB, Perrin JL, Blair SN (2006). Cardiorespiratory fitness is an independent predictor of hypertension incidence among initially normotensive healthy women. Am J Epidemiol, 163: 142-150. 16293717 10.1093/aje/kwj019

[25] Evenson KR, Stevens J, Cai J, Thomas R, Thomas O (2003). The effect of cardiorespiratory fitness and obesity on cancer mortality in women and men. Med Sci Sports Exerc, 35: 270-277. 12569216 10.1249/01.MSS.0000053511.02356.72

[26] Gronek P, Wielinski D, Cyganski P, Rynkiewicz A, Zając A, Maszczyk A, Gronek J, Podstawski R, Czarny W, Balko S, Ct Clark C, Celka R (2020). A Review of Exercise as Medicine in Cardiovascular Disease: Pathology and Mechanism. Aging Dis, 9;11(2):327-340. 32257545 10.14336/AD.2019.0516 PMC7069454

[27] Kodama S, Saito K, Tanaka S, Maki M, Yachi Y, Asumi M, Sugawara A, Totsuka K, Shimano H, Ohashi Y, Yamada N, Sone H (2009). Cardiorespiratory fitness as a quantitative predictor of all-cause mortality and cardiovascular events in healthy men and women: a meta-analysis. JAMA 301: 2024-2035. 19454641 10.1001/jama.2009.681

[28] Simpson RJ, Guy K (2010). Coupling aging immunity with a sedentary lifestyle: has the damage already been done?-a mini-review. Gerontology, 56(5): 449-458. 20029165 10.1159/000270905

[29] Haaland DA, Sabljic TF, Baribeau DA, Mukovozov IM, Hart LE (2008). Is regular exercise a friend or foe of the aging immune system? A systematic review. Clin J Sport Med, 18(6): 539-548. 19001887 10.1097/JSM.0b013e3181865eec

[30] Bartlett DB, Fox O, McNulty CL, Greenwood HL, Murphy L, Sapey E, et al (2016). Habitual physical activity is associated with the maintenance of neutrophil migratory dynamics in healthy older adults. Brain Behav Immun, 56: 12-20. 26928196 10.1016/j.bbi.2016.02.024 PMC4929133

[31] Njajou OT, Hsueh WC, Blackburn EH, Newman AB, Wu SH, Li R, et al (2009). Association between telomere length, specific causes of death, and years of healthy life in health, aging, and body composition, a population-based cohort study. J Gerontol A Biol Sci Med Sci, 64: 860-864. 19435951 10.1093/gerona/glp061 PMC2981462

[32] Sanders JL, Fitzpatrick AL, Boudreau RM, Arnold AM, Aviv A, Kimura M, et al (2012). Leukocyte telomere length is associated with noninvasively measured age-related disease: the cardiovascular health study. J Gerontol, 67(4): 409-416. 10.1093/gerona/glr173 PMC3309872 21934123

[33] Okuda K, Bardeguez A, Gardner JP, Rodriguez P, Ganesh V, Kimura, et al (2002). Telomere length in the newborn. Pediatr Res, 52(3): 377-381. 12193671 10.1203/00006450-200209000-00012

[34] Arai Y, Martin-Ruiz CM, Takayama M, Abe Y, Takebayashi T, Koyasu S, et al (2015). Inflammation, but not telomere length, predicts successful ageing at extreme old age: a longitudinal study of semi-supercentenarians. E Bio Medicine, 2(10): 1549-1558. 10.1016/j.ebiom.2015.07.029 PMC4634197 26629551

[35] Harley CB, Futcher AB, Greider CW (1990). Telomeres shorten during ageing of human fibroblasts. Nature, 345: 458-460. 2342578 10.1038/345458a0

[36] Cherkas LF, Hunkin JL, Kato BS, Richards JB, Gardner JP, Surdulescu GL, et al (2008). The association between physical activity in leisure time and leukocyte telomere length. Arch Intern Med, 168(2): 154-158. 18227361 10.1001/archinternmed.2007.39

[37] Deus LA, Sousa CV, Rosa TS, Filho JMS, Santos PA, Barbosa LD, et al. (2019). Heart rate variability in middle-aged sprint and endurance athletes. Physiol Behav, 205: 39-43. 30389479 10.1016/j.physbeh.2018.10.018

[38] Sousa CV, Aguiar SS, Santos PA, Barbosa LP, Knechtle B, Nikolaidis PT, et al. (2019). Telomere length and redox balance in master endurance runners: the role of nitric oxide. Exp Gerontol, 117: 113-118. 30481549 10.1016/j.exger.2018.11.018

[39] Ludlow AT, Zimmerman JB, Witkowski S, Hearn JW, Hatfield BD, Roth SM (2008). Relationship between physical activity level, telomere length, and telomerase activity. Med Sci Sports and Exerc, 40(10): 1764. 18799986 10.1249/MSS.0b013e31817c92aa PMC2581416

[40] Simões HG, Rosa TS, Sousa CV, Aguiar SDS, Motta-Santos D, Degens H, et al (2020). Does longer leukocyte telomere length and higher physical fitness protect master athletes from consequences of coronavirus (SARS-CoV-2) infection?. Front Sports Act Living, 2: 87. 33345078 10.3389/fspor.2020.00087 PMC7739763

[41] Chilton W, O’Brien B, Charchar F (2017). Telomeres, Aging and Exercise: Guilty by Association? Int J Mol Sci, 18(12):2573. 10.3390/ijms18122573 PMC5751176 29186077

[42] Denham J, Nelson CP, O’Brien BJ, Nankervis SA, Denniff M, Harvey JT, et al (2013). Longer leukocyte telomeres are associated with ultra-endurance exercise independent of cardiovascular risk factors. PLoS ONE, 8: e69377. 23936000 10.1371/journal.pone.0069377 PMC3729964

[43] Chilton WL, Marques FZ, West J, Kannourakis G, Berzins SP, O’Brien BJ et al (2014). Charchar, F.J. Acute exercise leads to regulation of telomere-associated genes and microrna expression in immune cells. PLoS ONE, 9: e92088 24752326 10.1371/journal.pone.0092088 PMC3994003

[44] Ludlow AT, Witkowski S, Marshall MR, Wang J, Lima LC, Guth LM, et al (2012). Chronic exercise modifies age-related telomere dynamics in a tissue-specific fashion. J Gerontol A Biol Sci Med Sci, 67(9): 911-926. 22389464 10.1093/gerona/gls002 PMC3436090

[45] Larbi A, Kempf J, Pawelec G (2007). Oxidative stress modulation and T cell activation. Exp Gerontol, 42(9): 852-858. 17604927 10.1016/j.exger.2007.05.004

[46] Harman D (2006). Free radical theory of aging: an update: increasing the functional life span. Ann NY Acad Sci, 1067: 10-21. 16803965 10.1196/annals.1354.003

[47] Harman D (1956). Aging: A Theory Based on Free Radical and Radiation Chemistry. J Gerontol, 11(3):298-300. 13332224 10.1093/geronj/11.3.298

[48] Bouzid MA, Filaire E, McCall A, Fabre C (2015). Radical oxygen species, exercise and aging: an update. Sports Med, 45(9): 1245-1261. 26119427 10.1007/s40279-015-0348-1

[49] Radak Z, Ishihara K, Tekus E, Varga C, Posa A, Balogh L, et al (2017). Exercise, oxidants, and antioxidants change the shape of the bell-shaped hormesis curve. Redox Biol, 12:285-90. 28285189 10.1016/j.redox.2017.02.015 PMC5345970

[50] Southam CM, Ehrlich J (1943). Effects of extract of western red-cedar heartwood on certain wood-decaying fungi in culture. Phytopathology, 33: 517-24.

[51] Csaba G (2019). Immunity and longevity. Acta Microbiol Immunol Hung, 66(1):1-17. 29968490 10.1556/030.65.2018.029

[52] Csaba G (2019). Hormesis and immunity: a review. Acta Microbiol Immunol Hung, 66(2): 155-168. 30014704 10.1556/030.65.2018.036

[53] Scott BR (2017). Small radiation doses enhance natural barriers to cancer. J Am Physicians Surg, 22(4): 105-110.

[54] Pingitore A, Lima GPP, Mastorci F, Quinones A, Iervasi G, Vassalle C (2015). Exercise and oxidative stress: potential effects of antioxidant dietary strategies in sports. Nutr, 31(7-8): 916-922. 10.1016/j.nut.2015.02.005 26059364

[55] Ji LL, Gomez-Cabrera MC, Vina J (2006). Exercise and hormesis: activation of cellular antioxidant signaling pathway. Ann NY Acad Sci, 1067: 425-435 16804022 10.1196/annals.1354.061

[56] Gomez-Cabrera MC, Domenech E, Vina J (2008). Moderate exercise is an antioxidant: Upregulation of antioxidant genes by training. Free Radic Biol Med, 44(2): 126-131. 18191748 10.1016/j.freeradbiomed.2007.02.001

[57] Campos M, Godson DL (2003). The effectiveness and limitations of immune memory: understanding protective immune responses. Int J Parasitol, 33(5-6): 655-661. 12782062 10.1016/s0020-7519(03)00066-3

[58] Littman DR (1987). The structure of the CD4 and CD8 genes. Annu Rev Immunol, 5(1): 561-584. 3109457 10.1146/annurev.iy.05.040187.003021

[59] Müller M, Carter S, Hofer MJ, Campbell IL (2010). The chemokine receptor CXCR3 and its ligands CXCL9, CXCL10 and CXCL11 in neuroimmunity - a tale of conflict and conundrum. Neuropathol Appl Neurobiol, 36(5): 368-387. 20487305 10.1111/j.1365-2990.2010.01089.x

[60] Nigam PK, Patra PK, Khodiar PK, Gual J (2011). A study of blood CD3+, CD4+, and CD8+ T cell levels and CD4+: CD8+ ratio in vitiligo patients. Indian J Dermatol Venereol Leprol, 77(1):111. 10.4103/0378-6323.74993 21220904

[61] Jin CH, Paik IY, Kwak YS, Jee YS, Kim JY (2015). Exhaustive submaximal endurance and resistance exercises induce temporary immunosuppression via physical and oxidative stress. J Exerc Rehabil, 11(4): 198. 26331134 10.12965/jer.150221 PMC4548676

[62] Linton P, Thoman ML (2001). T cell senescence. Front Biosci, 6(248): e61. 11171551 10.2741/linton

[63] Phillips AC, Burns VE, Lord JM (2007). Stress and exercise: Getting the balance right for aging immunity. Exerc Sport Sci Rev, 35(1): 35-39. 17211192 10.1097/jes.0b013e31802d7008

[64] Ouyang Q, Wagner WM, Wikby A, Walter S, Aubert G, Dodi AI, et al (2003). Large numbers of dysfunctional CD8+ T lymphocytes bearing receptors for a single dominant CMV epitope in the very old. J Clin Immunol, 23(4): 247-257. 12959217 10.1023/a:1024580531705

[65] Cosgrove C, Simpson R, Florida-James G, Whyte G, Guy K (2007). KLRG1 and CD57 are expressed on a greater proportion of CD8+ T lymphocytes from older subjects compared to those from younger subjects: OP68. Immunol, 120.

[66] Naylor K, Li G, Vallejo AN, Lee WW, Koetz K, Bryl E, et al (2005). The influence of age on T cell generation and TCR diversity. J Immunol, 174(11): 7446-7452. 15905594 10.4049/jimmunol.174.11.7446

[67] Ohkusu K, Du J, Isobe KI, Yi H, Akhand AA, Kato M, et al (1997). Protein kinase Ca-mediated chronic signal transduction for immunosenescence. J Immunol, 159: 2082-2084. 9278292

[68] Gupta S (2000). Molecular and biochemical pathways of apoptosis in lymphocytes from aged humans. Vaccine, 18: 1596-1601. 10689134 10.1016/s0264-410x(99)00492-2

[69] Castle SC, Uyemara K, Crawford W, Wong W, Klaustermeyer WB, Makinodan T (1999). Age-related impaired proliferation of peripheral blood mononuclear cells is associated with an increase in IL-10 and IL-12. Exper Geron, 34: 243-252. 10.1016/s0531-5565(98)00064-3 10363790

[70] Fernández-Lázaro D, Sanz Gómez N, Sánchez Serrano N, AlaouiSosse A, Aldea-Mansilla C (2020). Emergency Standardization for SARS-CoV-2 virus Diagnosis by Real- Time Reverse Transcription Polymerase Chain Reaction (RT-PCR) in a COVID-19 pandemic situation. Rev. Madrileña Salud Pública, 4(7): 1-11.

[71] Green DJ, Maiorana A, O'Driscoll G, Taylor R (2004). Effect of exercise training on endothelium-derived nitric oxide function in humans. J Physiol, 561(1): 1-25. 15375191 10.1113/jphysiol.2004.068197 PMC1665322

[72] Zbinden-Foncea H, Francaux M, Deldicque L, Hawley JA (2020). Does high cardiorespiratory fitness confer some protection against proinflammatory responses after infection by SARS-CoV-2?. Obesity, 28(8): 1378-1381. 32324968 10.1002/oby.22849 PMC7264673

[73] Nurmasitoh T (2015). Physical activities, exercises, and their effects to the immune system. JKKI, 7(2): 52-58.

[74] Smuder AJ, Morton AB, Hall SE, Wiggs MP, Ahn B, Wawrzyniak NR., et al (2019). Effects of exercise preconditioning and HSP72 on diaphragm muscle function during mechanical ventilation. J Cachexia Sarcopenia Muscle, 10(4): 767-781. 30972953 10.1002/jcsm.12427 PMC6711411

[75] Minuzzi LG, Rama L, Chupel MU, Rosado F, dos Santos JV, Simpson R, et al (2018). Effects of lifelong training on senescence and mobilization of T lymphocytes in response to acute exercise. Exerc Immunol Rev, 24:72-84. 29461967

[76] Pereira GB, Prestes J, Tibana RA, Shiguemoto GE, Navalta J, Perez SEA (2012). Acute resistance training affects cell surface markers for apoptosis and migration in CD4+ and CD8+ lymphocytes. Cell Immunol, 279(2): 134-139. 23246503 10.1016/j.cellimm.2012.11.002

[77] Simpson RJ (2011). Aging, persistent viral infections, and immunosenescence: can exercise" make space"?. Exerc Sport Sci Rev, 39(1): 23-33. 21088603 10.1097/JES.0b013e318201f39d

[78] Friedman RA, Navalta JW, Fedor EA, Kell HB, Lyons TS, Arnett SW, et al (2012). Repeated high-intensity Wingate cycle bouts influence markers of lymphocyte migration but not apoptosis. Appl Physiol Nutr Metabol, 37(2): 241-246. 10.1139/h11-156 22380726

[79] Uinarni H, Carmelita AB, Trisia A, Prayudhistya BKA, Ahmad H (2020). Cortisol, IL-6, TNF Alfa, Leukocytes and DAMP on Exercise. Sys Rev Pharm, 11(6): 474 485.

[80] American College of Sports Medicine (2009). American College of Sports Medicine position stand. Progression models in resistance training for healthy adults. Med Sci Sports Exerc, 41(3): 687-708. 19204579 10.1249/MSS.0b013e3181915670

[81] Martin SA, Pence BD, Woods JA (2009). Exercise and respiratory tract viral infections. Exerc Sport Sci Rev, 37(4): 157. 19955864 10.1097/JES.0b013e3181b7b57b PMC2803113

[82] Grande AJ, Keogh J, Silva V, Scott AM (2020). Exercise versus no exercise for the occurrence, severity, and duration of acute respiratory infections. Cochrane Database Syst Rev, 2020,4(4):CD010596. 10.1002/14651858.CD010596.pub3 PMC7127736 32246780

[83] Singh VK, Mehrotra S, Agarwal SS (1999). The paradigm of Th1 and Th2 cytokines. Immunol Res, 20.3: 147-161. 10580639 10.1007/BF02786470

[84] Kostka T, Berthouze SE, Lacour J, Bonnefoy M (2000). The symptomatology of upper respiratory tract infections and exercise in elderly people. Med Sci Sports Exerc, 32(1):46-51. 10647528 10.1097/00005768-200001000-00008

[85] Wong CM, Lai HK, Ou CQ, Ho SY, Chan KP, Thach TQ, et al (2008). Is exercise protective against influenza-associated mortality? PLoS One, 3(5):e2108. 18461130 10.1371/journal.pone.0002108 PMC2329855

[86] Davis JM, Murphy EA, Brown AS, Carmichael MD, Ghaffar A, Mayer EP (2004). Effects of oat beta-glucan on innate immunity and infection after exercise stress. Med Sci Sports Exerc, 36(8):1321-7. 15292739 10.1249/01.mss.0000135790.68893.6d

[87] Lowder T, Padgett DA, Woods JA (2005). Moderate exercise protects mice from death due to influenza virus. Brain BehavImmun, 19(5):377-80. 10.1016/j.bbi.2005.04.002 15922557

[88] Ekblom B, Ekblom O, Malm C (2006). Infectious episodes before and after a marathon race. Scand J Med Sci Sports, 16(4):287-93. 16895535 10.1111/j.1600-0838.2005.00490.x

[89] Chubak J, McTiernan A, Sorensen B, Wener MH, Yasui Y, Velasquez M, et al (2006). Moderate-intensity exercise reduces the incidence of colds among postmenopausal women. Am J Med, 119(11):937-42. 17071161 10.1016/j.amjmed.2006.06.033

[90] Weidner TG, Cranston T, Schurr T, Kaminsky LA (1998). The effect of exercise training on the severity and duration of a viral upper respiratory illness. Med Sci Sports Exerc, 30(11): 1578-1583. 9813869 10.1097/00005768-199811000-00004

[91] Momesso dos Santos CM, Sato FT, Cury-Boaventura MF, Guirado-Rodrigues SH, Caçula KG, Gonçalves Santos CC, et al (2015). Effect of Regular Circus Physical Exercises on Lymphocytes in Overweight Children. PLoS ONE, 10(3). 10.1371/journal.pone.0120262 PMC4380297 25826263

[92] Woods JA, Hutchinson NT, Powers SK, Roberts WO, Gomez-Cabrera MC, Radak Z, et al (2020). The COVID-19 pandemic and physical activity. Sports Medi Health Sci, 2(2): 55-64. 10.1016/j.smhs.2020.05.006 PMC7261095 34189484

[93] Geffken D, Cushman M, Burke G, Polak J, Sakkinen P, Tracy R (2001). Association between physical activity and markers of inflammation in a healthy elderly population. Am J Epidemiol, 153:242-50. 11157411 10.1093/aje/153.3.242

[94] Colbert LH, Visser M, Simonsick EM, Tracy RP, Newman AB, Kritchevsky SB, et al (2004). Physical activity, exercise, and inflammatory markers in older adults: findings from the Health, Aging and Body Composition Study. J Am Geriatr Soc, 52:1098-104. 15209647 10.1111/j.1532-5415.2004.52307.x

[95] Salamat KM, Azarbayjani MA, Yusof A, Dehghan F (2016). The response of pre-inflammatory cytokines factors to different exercises (endurance, resistance, concurrent) in overweight men. Alexandria J Med, 52(4): 367-370.

[96] Long JE, Ring C, Drayson M, Bosch J, Campbell JP, Bhabra J, et al (2012). Vaccination response following aerobic exercise: can a brisk walk enhance antibody response to pneumococcal and influenza vaccinations? Brain Behav Immun, 26(4): 680-687. 22386744 10.1016/j.bbi.2012.02.004

[97] Edwards KM, Burns VE, Allen LM, McPhee JS, Bosch JA, Carroll D, et al (2007). Eccentric exercise as an adjuvant to influenza vaccination in humans. Brain Behav Immun, 21(2): 209-217. 16824730 10.1016/j.bbi.2006.04.158

[98] Edwards KM, Campbell JP (2011). Acute exercise as an adjuvant to influenza vaccination. Am J Lifestyle Med, 5(6): 512-517.

[99] Kohut ML, Arntson BA, Lee W, Rozeboom K, Yoon KJ, Cunnick JE, et al (2004). Moderate exercise improves antibody response to influenza immunization in older adults. Vaccine, 22(17-18): 2298-2306. 15149789 10.1016/j.vaccine.2003.11.023

[100] Kohut ML, Cooper MM, Nickolaus MS, Russell DR, Cunnick JE (2002). Exercise and psychosocial factors modulate immunity to influenza vaccine in elderly individuals. J Gerontol A Biol Sci Med Sci, 57(9): M557-M562. 12196490 10.1093/gerona/57.9.m557

[101] Woods JA, Keylock KT, Lowder T, Vieira VJ, Zelkovich W, Dumich S, et al (2009). Cardiovascular exercise training extends influenza vaccine seroprotection in sedentary older adults: the immune function intervention trial. J Am Geriat Soc, 57(12): 2183-2191. 20121985 10.1111/j.1532-5415.2009.02563.x

[102] Andrew MK, Bowles SK, Pawelec G, Haynes L, Kuchel GA, McNeil SA et al (2019). Influenza Vaccination in Older Adults: Recent Innovations and Practical Applications. Drugs Aging, 36: 29-37. 30411283 10.1007/s40266-018-0597-4

[103] Franceschi C, Bonafè M, Valensin S, Olivieri F, De Luca M, Ottaviani E, et al (2000).Inflamm-aging. An evolutionary perspective on immunosenescence. Ann NY AcadSci, 908: 244-254 10.1111/j.1749-6632.2000.tb06651.x 10911963

[104] De Martinis M, Franceschi C, Monti D, Ginaldi L (2005). Inflamm-ageing and lifelong antigenic load as major determinants of ageing rate and longevity. FEBS Lett, 579(10): 2035-2039. 15811314 10.1016/j.febslet.2005.02.055

[105] Morrisette-Thomas V, Cohen AA, Fülöp T, Riesco É, Legault V, Li Q, et al (2014). Inflamm-aging does not simply reflect increases in pro-inflammatory markers. Mech Ageing Dev, 139: 49-57. 25011077 10.1016/j.mad.2014.06.005 PMC5881904

[106] Flynn MG, Markofski MM, Carrillo AE (2019). Elevated inflammatory status and increased risk of chronic disease in chronological aging: inflamm-aging or inflamm-inactivity? Aging Dis, 10(1): 147. 30705775 10.14336/AD.2018.0326 PMC6345337

[107] Simpson RJ (2016). Aging and inflammation: directing traffic through physical activity. Brain BehavImmun, 56: 10-11. 10.1016/j.bbi.2016.05.015 27223097

[108] Dixit S (2020). Can moderate intensity aerobic exercise be an effective and valuable therapy in preventing and controlling the pandemic of COVID-19? Med Hypotheses, 143: 109854. 32464492 10.1016/j.mehy.2020.109854 PMC7237357

[109] Gronek P, Haas AN, Czarny W, Podstawski R, do Santos Delabary M, Clark CC, et al (2021). The mechanism of physical activity-induced amelioration of Parkinson’s disease: A narrative review. Aging Dis, 12(1): 192. 33532136 10.14336/AD.2020.0407 PMC7801266

[110] Woods JA, Hutchinson NT, Powers SK, Roberts WO, Gomez-Cabrera MC, Radak Z, et al (2020). The COVID-19 pandemic and physical activity. SMHS, 2(2): 55-64. 34189484 10.1016/j.smhs.2020.05.006 PMC7261095

[111] Brandt C, Pedersen BK (2010). The role of exercise-induced myokines in muscle homeostasis and the defense against chronic diseases. J Biomed Biotechnol, 520258. 10.1155/2010/520258 PMC2836182 20224659

[112] Woods JA, Wilund KR, Martin SA, Kistler BM (2012). Exercise, inflammation and aging. Aging Dis, 3(1):130-40. 22500274 PMC3320801

[113] Nieman DC, Henson DA, Austin MD, Sha W (2011). Upper respiratory tract infection is reduced in physically fit and active adults. Br J Sports Med, 45: 987-992. 21041243 10.1136/bjsm.2010.077875

[114] León-Latre M, Moreno-Franco B, Andrés-Esteban EM, Ledesma M, Laclaustra M, Alcalde V, et al (2014). Sedentary lifestyle and its relation to cardiovascular risk factors, insulin resistance and inflammatory profile. Rev Esp Cardiol (Engl Ed), 67(6): 449-455. 24863593 10.1016/j.rec.2013.10.015

[115] Yates LB, Djoussé L, Kurth T, Buring JE, Gaziano JM (2008). Exceptional longevity in men: modifiable factors associated with survival and function to age 90 years. Arch of Intern Med, 168(3): 284-290. 18268169 10.1001/archinternmed.2007.77

[116] Beavers KM, Brinkley TE, Nicklas BJ (2010). Effect of exercise training on chronic inflammation. Clinica Chimica Acta, 411(11-12): 785-793. 10.1016/j.cca.2010.02.069 PMC3629815 20188719

[117] Nicklas BJ, You T, Pahor M (2005). Behavioural treatments for chronic systemic inflammation: effects of dietary weight loss and exercise training. CMAJ, 172(9): 1199-1209. 15851714 10.1503/cmaj.1040769 PMC557073

[118] Starkie R, Ostrowski SR, Jauffred S, Febbraio M, Pedersen BK (2003). Exercise and IL-6 infusion inhibit endotoxin-induced TNF-alpha production in humans. FASEB J, 17:884-886. 12626436 10.1096/fj.02-0670fje

[119] da Luz Scheffer D, Latini A (2020). Exercise-induced immune system response: Anti-inflammatory status on peripheral and central organs. Biochim Biophys Acta Mol Basis Dis, 1866(10): 165823. 32360589 10.1016/j.bbadis.2020.165823 PMC7188661

[120] Adamson S, Kavaliauskas M, Yamagishi T, Phillips S, Lorimer R., Babraj J. (2019). Extremely short duration sprint interval training improves vascular health in older adults. Sport Sci Health, 15: 123-131.

[121] Scartoni FR, Sant’Ana LDO, Murillo-Rodriguez E, Yamamoto T, Imperatori C, Budde H, et al. (2020). Physical exercise and immune system in the elderly: implications and importance in COVID-19 pandemic period. Front Psychol, 11: 3215. 10.3389/fpsyg.2020.593903 PMC7711129 33329256

[122] Mullins AL, Van Rosendal SP, Briskey DR, Fassett RG, Wilson GR, Coombes JS (2013). Variability in oxidative stress biomarkers following a maximal exercise test. Biomarkers, 18: 446-454. 23862764 10.3109/1354750X.2013.810668

[123] Winett RA, Ogletree AM. (2019). Evidence-based, high-intensity exercise and physical activity for compressing morbidity in older adults: a narrative review. Innov Aging, 3(2): igz020. 31380470 10.1093/geroni/igz020 PMC6658199

[124] Scherr J, Wolfarth B, Christle JW, Pressler A, Wagenpfeil S, Halle M (2013). Associations between Borg’s rating of perceived exertion and physiological measures of exercise intensity. Eur J Appl Physiol, 113(1): 147-155. 22615009 10.1007/s00421-012-2421-x

[125] Ost M, Coleman V, Kasch J, Klaus S (2016). Regulation of myokine expression: Role of exercise and cellular stress. Free Radic Biol Med, 98: 78-89. 26898145 10.1016/j.freeradbiomed.2016.02.018

[126] Lee DC, Brellenthin AG, Thompson PD, Sui X, Lee IM, Lavie CJ (2017). Running as a key lifestyle medicine for longevity. Prog Cardiovasc Dis, 60(1): 45-55. 28365296 10.1016/j.pcad.2017.03.005

[127] Chakravarty EF, Hubert HB, Lingala VB, Fries JF (2008). Reduced disability and mortality among aging runners: a 21-year longitudinal study. Arch Intern Med, 168(15): 1638-1646. 18695077 10.1001/archinte.168.15.1638 PMC3175643

[128] Oja P, Kelly P, Pedisic Z, Titze S, Bauman A, Foster C, et al (2017). Associations of specific types of sports and exercise with all-cause and cardiovascular-disease mortality: a cohort study of 80 306 British adults. Bri J Sports Med, 51(10): 812-817. 10.1136/bjsports-2016-096822 27895075

[129] Pedisic Z, Shrestha N, Kovalchik S, Stamatakis E, Liangruenrom N, Grgic J, et al (2020). Is running associated with a lower risk of all-cause, cardiovascular and cancer mortality, and is the more the better? A systematic review and meta-analysis. Br J Sports Med, 54(15): 898-905. 31685526 10.1136/bjsports-2018-100493

[130] Schnohr P, O’Keefe JH, Marott JL, Lange P, Jensen GB (2015). Dose of jogging and long-term mortality: the Copenhagen City Heart Study. J Am Coll Cardiol, 65(5): 411-419. 25660917 10.1016/j.jacc.2014.11.023

[131] Dai S, Carroll DD, Watson KB, Paul P, Carlson SA, Fulton JE (2015). Participation in types of physical activities among US adults—National Health and Nutrition Examination Survey 1999-2006. J Phys Act Health, 12(s1): S128-S140. 26083795 10.1123/jpah.2015-0038 PMC4487906

[132] Sergio G (2008). Exploring the complex relations between inflammation and aging (inflamm-aging): anti-inflamm-aging remodelling of inflamm-aging, from robustness to frailty. Inflamm Res, 57(12): 558-563. 19109735 10.1007/s00011-008-7243-2

[133] Cooper DM, Radom-Aizik S, Schwindt C, Zaldivar F Jr (2007). Dangerous exercise: lessons learned from dysregulated inflammatory responses to physical activity. J App Physiol, 103(2): 700-709. 10.1152/japplphysiol.00225.2007 17495117

[134] Turner JE, Bennett SJ, Campbell JP, Bosch JA, Aldred S, Griffiths HR (2013). The antioxidant enzyme peroxiredoxin-2 is depleted in lymphocytes seven days after ultra-endurance exercise. Free Radic Res, 47(10): 821-828. 23889121 10.3109/10715762.2013.828836

[135] Kakanis MW, Peake J, Hooper S, Gray B, Marshall-Gradisnik S (2010). The open window of susceptibility to infection after acute exercise in healthy young male elite athletes. J Sci Med Sport, 13:e85-e86. 20839496

[136] Stout RD, Jiang C, Matta B, Tietzel I, Watkins SK, Suttles J (2005). Macrophages sequentially change their functional phenotype in response to changes in microenvironmental influences. J Immunol, 175: 342-349. 15972667 10.4049/jimmunol.175.1.342

[137] Mosser DM (2003). The many faces of macrophage activation. J Leukoc Biol, 73: 209-212. 12554797 10.1189/jlb.0602325

[138] Hughes JE, Srinivasan S, Lynch KR, Proia RL, Ferdek P, Hedrick CC (2008). Sphingosine-1-phosphate induces an antiinflammatory phenotype in macrophages. Circ Res, 102(8): 950-958. 18323526 10.1161/CIRCRESAHA.107.170779 PMC2875063

[139] Ohashi W, Hattori K, Hattori Y (2015). Control of macrophage dynamics as a potential therapeutic approach for clinical disorders involving chronic inflammation. J Pharmacol Exp Ther, 354(3): 240-250. 26136420 10.1124/jpet.115.225540

[140] Pedersen BK (2011). Exercise-induced myokines and their role in chronic diseases. Brain Behav Immun, 25(5): 811-816. 21354469 10.1016/j.bbi.2011.02.010

[141] So B, Kim HJ, Kim J, Song W (2014). Exercise-induced myokines in health and metabolic diseases. Integr Med Res, 3(4): 172-179. 28664094 10.1016/j.imr.2014.09.007 PMC5481763

[142] Ostrowski K, Rohde T, Asp S, Schjerling P, Pedersen BK (1999). Pro-and anti-inflammatory cytokine balance in strenuous exercise in humans. J Physiol, 515(1): 287-291. 9925898 10.1111/j.1469-7793.1999.287ad.x PMC2269132

[143] Steensberg A, Fischer CP, Keller C, Moller K, Pedersen BK (2003). IL-6 enhances plasma IL-1ra, IL-10, and cortisol in humans. Am J Physiol, 285(2): E433-E437, 2003. 10.1152/ajpendo.00074.2003 12857678

[144] Sellami M, Bragazzi NL, Aboghaba B, Elrayess MA (2021). The impact of acute and chronic exercise on immunoglobulins and cytokines in elderly: insights from a critical review of the literature. Front Immunol, 14;12:631873. 10.3389/fimmu.2021.631873 PMC8079972 33936044

[145] Divella R, De Luca R, Abbate I, Naglieri E, Daniele A (2016). Obesity and cancer: the role of adipose tissue and adipocytokines-induced chronic inflammation. J Cancer, 7(15): 2346-2359. 27994674 10.7150/jca.16884 PMC5166547

[146] Vieira VJ, Valentine RJ, Wilund KR, Antao N, Baynard T, Woods J A (2009). Effects of exercise and low-fat diet on adipose tissue inflammation and metabolic complications in obese mice. Am J Physiol Endocrinol Metab, 296(5): E1164-E1171. 19276393 10.1152/ajpendo.00054.2009 PMC2681303

[147] Bruun JM, Helge JW, Richelsen B, Stallknecht B (2006). Diet and exercise reduce low-grade inflammation and macrophage infiltration in adipose tissue but not in skeletal muscle in severely obese subjects. Am J Physiol Endocrinol Metab, 290(5):E961-7. 16352667 10.1152/ajpendo.00506.2005

[148] Forsythe LK, Wallace JM, Livingstone MBE (2008). Obesity and inflammation: the effects of weight loss. Nutr Res Rev, 21(2): 117-133. 19087366 10.1017/S0954422408138732

[149] Divella R, Mazzocca A, Daniele A, Sabbà C, Paradiso A (2019). Obesity, nonalcoholic fatty liver disease and adipocytokines network in promotion of cancer. Intern J Biol Sci, 15(3): 610. 10.7150/ijbs.29599 PMC6367583 30745847

[150] Dorneles GP, Haddad DO, Fagundes VO, Vargas BK, Kloecker A, Romão PRT, et al (2016). High intensity interval exercise decreases IL-8 and enhances the immunomodulatory cytokine interleukin-10 in lean and overweight-obese individuals. Cytokine, 77: 1-9. 26476404 10.1016/j.cyto.2015.10.003

[151] Kern P, Saghizadeh M, Ong J, Bosch R, Deem R, Simsolo R (1995). The expression of tumor necrosis factor in human adipose tissue. Regulation by obesity, weight loss, and relationship to lipoprotein lipase. J Clin Invest, 95(5): 2111-9. 7738178 10.1172/JCI117899 PMC295809

[152] Magliulo L, Bondi D, Pini N, Marramiero L, Di Filippo ES (2021). The wonder exerkines—novel insights: a critical state-of-the-art review. Mol Cell Biochem, 1-9. 10.1007/s11010-021-04264-5 PMC8755664 34554363

[153] Pedersen BK (2009). The diseasome of physical inactivity-and the role of myokines in muscle-fat cross talk. J Physiol, 587(23): 5559-5568. 19752112 10.1113/jphysiol.2009.179515 PMC2805368

